# Antisynthetase Syndrome-Associated Interstitial Lung Disease: Monitoring of Immunosuppressive Treatment Effects by Chest Computed Tomography

**DOI:** 10.3389/fmed.2020.609595

**Published:** 2021-01-25

**Authors:** Peter Korsten, Jan-Gerd Rademacher, Linn Riedel, Eva-Maria Schnitzler, Ulrike Olgemöller, Cornelia Sabine Seitz, Jens Schmidt, Jörg Larsen, Radovan Vasko

**Affiliations:** ^1^Department of Nephrology and Rheumatology, University Medical Center Goettingen, Goettingen, Germany; ^2^Institute of Diagnostic and Interventional Radiology, University Medical Center Goettingen, Goettingen, Germany; ^3^Department of Cardiology and Pulmonology, University Medical Center Goettingen, Goettingen, Germany; ^4^Department of Dermatotology, Allergology, and Venereology, University Medical Center Goettingen, Goettingen, Germany; ^5^Department of Neurology, University Medical Center Goettingen, Goettingen, Germany

**Keywords:** antisynthetase syndrome, interstitial lung disease, immunosuppressive agents, inflammatory myopathies, myositis

## Abstract

**Background:** Antisynthetase syndrome (ASyS) is a rare autoimmune disease characterized by inflammatory myopathy, arthritis, fever, and interstitial lung disease (ILD). Pulmonary involvement in ASyS significantly increases morbidity and mortality and, therefore, requires prompt and effective immunosuppressive treatment. Owing to the rarity of ASyS, limited data exists on progression and prognosis of ILD under immunosuppression.

**Objectives:** The objective of the study was to evaluate the radiological progression and outcome measures of ILD with immunosuppressive therapy in patients with ASyS.

**Methods:** Twelve patients with ASyS-associated ILD (ASyS-ILD) were included. Demographic and clinical data, including organ involvement, pulmonary function tests (PFT), laboratory parameters, imaging studies, and treatment regimens were retrospectively analyzed from routinely collected data. The extent of ground glass opacities, fibrotic changes and honeycombing was analyzed and scored using high-resolution chest computed tomography (HRCT) scans. HRCT findings were compared between baseline and follow-up examinations. In addition, patients were stratified depending on whether they had received rituximab (RTX) or not.

**Results:** Pulmonary function tests revealed stable lung function and follow-up HRCT scans showed an improvement of radiological alterations in the majority of ASyS patients under immunosuppressive therapy. We did not detect significant differences between the RTX- and non-RTX-treated groups, but the RTX-treated patients more frequently had myositis and relapsing disease.

**Conclusions:** Radiographic alterations in ASyS-associated ILD respond to immunosuppressive treatment. RTX is a feasible treatment option with similar clinical and radiographic outcomes in patients with relapsing disease and clinically apparent myositis.

## Introduction

Antisynthetase syndrome (ASyS) is a rare autoimmune disease, belonging to the idiopathic inflammatory myopathies (IIM) (1). Due to frequent extramuscular manifestations, including fever, Raynaud's syndrome, arthritis, mechanic's hands and interstitial lung disease (ILD) (2, 3), ASyS is classified among the overlap myositis (4). Specific antibodies (abs) directed against different aminoacyl-tRNA synthetases (ARS) are the serological markers of ASyS. Anti-Jo1 abs are the most frequently detected ARS abs, and they are observed in up to 30% of patients with IIMs, whereas other ARS abs, such as anti-PL-7, anti-PL-12, anti-EJ, and anti-OJ, are less frequently detected (5). The classical clinical triad (arthritis, myositis, and ILD) can be observed in up to 90% of patients, but it is not always present in the early stages of the disease (6). The overall prognosis depends on the extent of organ involvement and on the occurrence of malignancies, which are, however, less common than in other IIMs subsets (7). ASyS-ILD is the most important prognostic factor in these patients and lung involvement is associated with an increased risk of mortality, thus requiring prompt immunosuppressive treatment (7, 8). To date, there is no standardized treatment for AsyS, and different therapeutic protocols have been adopted from other forms of inflammatory myositis (9). In most cases, glucocorticoids (GC) in combination with other immunosuppressive agents, such as cyclosporine (CsA) (10), methotrexate (MTX), azathioprine (AZA), or mycophenolate mofetil (MMF), cyclophosphamide (CYC), and rituximab (RTX) have been used in ASyS (9, 11).

In this single-center cohort study, we studied the effect of immunosuppression on high-resolution chest computed tomography (HRCT) findings in the course of ASyS-ILD, focusing particular on RTX.

## Patients and Methods

This retrospective observational study used routinely collected clinical data in patients with ASyS. The clinical care of patients with IIMs is organized in an interdisciplinary way among the Departments of Rheumatology, Neurology, Pulmonology, Dermatology, and Neuropathology, and relies on the use of standardized operating procedures. Management decisions are discussed and evaluated in multidisciplinary case conferences held on a monthly basis (12).

### Patient Identification

All patients fulfilling at least two or more clinical findings consistent with ASyS (arthritis, myositis, ILD, Raynaud's phenomenon, mechanic's hands, or ARS abs) were recruited from the University Medical Center Goettingen and their medical records were independently reviewed by three investigators who extracted the data (PK, JGR and LR). Additional patients were identified by the analysis of positively detected ARS abs at our DIN:ISO 2001 certified autoimmune laboratory.

Tests for myositis-associated (MAA) and myositis-specific (MSA) antibodies including Mi-2 alpha, Mi-2 beta, TIF1 gamma, MDA5, NXP2, SAE1, Ku, PM-Scl100, PM-Scl75, Jo-1, SRP, PL-7, PL-12, EJ, OJ, and Ro-52 were performed using the 16 Ag EUROLINE Blot (Euroimmun AG, Lübeck, Germany). The presence of additional antibodies was examined using the Elia™ SymphonyS test assay (Thermo Fisher Scientific, Waltham, MA, USA), which screens for, among others, the presence of anti-SSA antibodies (60 and 52 kDa). Anti-SSA-antibodies have been reported in about 50% of patients with incomplete forms of ASyS (13). Patients were stratified according to treatment into two groups: patients which never received RTX and a second group which received RTX in the course of ASyS-ILD.

### Data Assessment and Outcome Measures

Demographic data and clinical parameters were retrieved from patients' medical records. We evaluated the presence and spectrum of specific abs, organ involvement, laboratory parameters, pulmonary function tests (PFT), and imaging procedures in each patient. Histologic evidence of organ involvement was recorded, if available. As outcome measures for pulmonary involvement, we assessed the alteration of lung parenchyma on HRCT as well as PFTs before and during treatment. To assess the effect of immunosuppression, we recorded all patients' individual therapies used between the first and any subsequent follow-up HRCT scans. Progressive ILD was defined as worsening on imaging studies or worsening of PFT [at least a 10% decline of forced vital capacity (FVC) or at least a 15% decline of diffusion capacity for carbon monoxide (DLCO)].

### HRCT Scanning and Interpretation

Baseline and follow-up HRCT scans were obtained with 4-, 16-, 64-, and 128-slice scanners from 2011–2019 at the same institute. The scans were interpreted and scored independently and blinded to patient identity and clinical details by a senior registrar-level radiology resident (EMS) and a board-certified thoracic radiologist (JL) with 4 and 22 years of experience, respectively.

The analysis of HRCT patterns was performed in line with the CT-evaluation used in the *Scleroderma Lung Study* by Goldin et al. (14): during the initial assessment, the presence or absence of other important comorbidities was noted. For comprehensive scoring, each lung was divided into three zones: upper (lung apex to carina), middle (carina to inferior pulmonary veins terminus) and inferior (inferior pulmonary veins to lung bases), creating a total of six zones. The following lung findings were assessed and quantified: ground-glass opacities (GGO), fibrotic changes, interlobular changes and bronchiectasis (FIB), honey combing or subpleural cysts (HC). In baseline and follow-up data sets, the degree of abnormality in each lung zone was scored from 0to 4 (where 0 indicates absence, 1 = 1–25% involvement, 2 = 26–50%, 3 = 51–75% and 4 = 76–100%), as described previously (14). An example of the HRCT evaluation and terminology is presented in Supplementary Figure 1. For each study patient, baseline and follow-up measurements were determined using the overall mean of the entire lung for each abnormal parameter.

### Statistical Methods

Demographic data of the study population were analyzed by descriptive statistics. The Shapiro-Wilk test was used for testing normal (Gaussian) distribution. Parametric between-group-comparisons were performed with either the Student's *t*-test for paired data (two groups) or mixed effects analysis with Tukey test as *post-hoc* analysis for multiple comparisons (more than two groups). Mixed effects analysis was used because repeated measures analysis of variance (ANOVA) cannot handle missing values. We analyzed the data instead by fitting a mixed model. This mixed model uses a compound symmetry covariance matrix and is fit using Restricted Maximum Likelihood (REML). In the absence of missing values, this method gives the same *P*-values and multiple comparisons tests as repeated measures ANOVA. In the presence of missing values (missing completely at random), the results can be interpreted like repeated measures ANOVA. Geisser-Greenhouse correction was used.

Non-parametric between-group-comparisons were performed with Fisher's exact test. The interrater agreement of HRCT scores between the two radiologists was assessed by the weighted kappa statistic. Values below 0.20 were considered as poor, 0.21–0.40 fair, 0.41–0.60 moderate, 0.61–0.80 good, and 0.81–1.0 as very good agreement. *P* < 0.05 were considered statistically significant. Data analyses were performed with GraphPad Prism (version 8.4.0 for MacOS, GraphPad Software, San Diego, CA, USA) or STATA (STATA/MP version 16.1 for Windows, Stata Corp LLC, College Station, TX, USA).

## Results

### Patient Cohort

We identified 22 patients with positive ARS abs. One additional patient met clinical criteria (ILD, arthritis, mechanic's hands, fever) for ASyS but tested positive for anti-RO52 abs only. Testing for antinuclear antibodies in this patient revealed a cytoplasmic fine-speckled staining pattern.

Of the 22 patients with positive ARS abs, nine were excluded because they did not have ILD; one patient was not eligible due to incomplete data. Therefore, a total number of 12 patients (eight female and four male patients) was included in the final analysis (Supplementary Figure 2). Of these, seven received RTX, five did not receive RTX.

Median follow-up time was 31 (6–156) months. Demographic and clinical characteristics are presented in Table 1. There were no differences between the RTX ever- vs. RTX never-groups with the exception of clinically significant myositis, which was only present in the RTX-treated patients.

**Table 1:** Demographic and clinical characteristics of the patient cohort.

	**RTX ever *N* = 7**	**RTX never *N* = 5**	***p*-Value**
Median age at diagnosis (range)	45 years (32–62)	39 years (22–53)	*p* = 0.39
Gender	5 females 2 males	3 females 2 males	*P* > 0.99
Median length of follow-up^*^	17 months (7–35)	32 months (1–156)	*p* = 0.21
**Comorbidities**	5 (71.4%)	4 (80%)	
Arterial hypertension	1 (20%)	1 (25%)	*p* = 0.24
Diabetes mellitus	0	0	–
Stroke	1 (20%)	1 (25%)	*p* > 0.99
Malignancy	2 (40%; 1 Ovarian-cancer, 1 Breast-cancer)	1 (25%; 1 Breast-cancer)	*p* > 0.99
Smoking status			
Never smoked	5 (71.4%)	0	*p* > 0.99
Past smoker	0	4 (100%)	*p* = 0.42
Current smoker	2 (28.6%)	1 (25%)	*p* > 0.99
**Antisynthetase antibodies**	6 (86.7%)	5 (100%)	
Anti-tRNA-synthetase (Jo1)	5 (83%)	5 (100%)	*p* = 0.47
Anti-hRNA-synthetase (PL7)	1 (17%%)	0	*p* > 0.99
**Other antibodies**			
Anti-RO52	2 (28.6%)	0	*p* = 0.47
Anti-SS-A (Ro60)	4 (57.2%)	4 (80%)	*p* = 0.58
**Pulmonary function at baseline (% predicted and standard deviation)**			
Mean FVC	76.3 ± 22.3	90 ± 5.66	*p* = 0.44
Mean DLCO SB	70.3 ± 10.6	99.5 ± 30.4	*p* = 0.06
**Organ manifestations**			
ILD	7 (100%)	5 (100%)	*p* > 0.99
Arthritis	5 (71.4%)	2 (40%)	*p* = 0.56
Mechanic's hands	2 (28.6%)	0	*p* = 0.47
Raynaud's phenomenon	3 (42.9%)	1 (20%)	*p* = 0.58
Fever	2 (28.6%)	1 (20%)	*p* > 0.99
**Myositis**	**5 (71.4%)**	**0**	***p*** **<** **0.05**

DLCO, diffusion capacity for carbon monoxide; dsDNA, double-stranded deoxyribonucleic acid; FVC, forced vital capacity; ILD, interstitial lung disease; RTX, rituximab.

* *Follow-up denotes interval between CT investigation CT1 and CT2. Bold values indicates statistically significant*.

### Spectrum of Specific Antibodies in the Study Cohort

Anti-Jo-1 abs were present in 10 of 12 patients (83.3%). In addition, 10 of these 12 patients also tested positive for anti-SSA (detected either by ELiA, which recognizes anti-Ro52/Ro60, or anti-RO52 detected by Immunoblot; see Methods for details).

### Organ Manifestations

Arthritis (joint pain and swelling) was clinically evident in 7 patients (58%), mainly involving the hands. Myositis [defined as myalgia accompanied by elevation of creatinine kinase (CK), consistent muscle biopsy, or compatible findings on magnetic resonance imaging] was present in five patients (5/12; 41.6%) and was the second most common manifestation.

Creatine kinase levels during follow-up remained stable or improved in all cases. Two of the patients had a remote history of breast cancer, one patient had received a diagnosis of ASyS during pregnancy related to ovarian cancer.

### Immunosuppressive Treatment

Ten individuals received glucocorticoids (GC) while seven patients were treated with RTX during the study period. The reasons for RTX initiation were ILD progression in four patients (4/7, 57.1%), ILD at the time of ASyS diagnosis in two patients (2/7, 28.6%) and treatment of concomitant anti-CCP antibody positive rheumatoid arthritis in one patient (14.3%). Equally, relapsing disease with ILD flares and clinically apparent myositis in 5/12 (41.6%) patients led to RTX initiation. Six patients (6/12, 50%) received azathioprine (AZA). Less frequently used immunosuppressants were MTX in three and MMF in two patients. CYC (one patient), leflunomide (LEF; one patient), hydroxychloroquine (HCQ; one patient), and adalimumab (ADA; one patient) were used infrequently. The individual therapeutic regimens are presented in Table 2.

**Table 2:** Immunosuppressive treatments and outcomes of antisynthetase-associated interstitial lung disease.

**Patient**	**Therapy**				**Outcome**
	**1st line**	**2nd/3rd line**	**GC**	**Reason for RTX**	
RTX 1	RTX remission induction 2 × 1 g 4 w after Dx	MTX maintenance therapy 15 mg qw	5 mg qd	ILD at Dx, relapsing myositis	Improvement of ILD, stable lung function. Fatigue, no joint/muscle complaints
RTX 2	RTX remission induction 2 × 1 g 4 w after Dx, 2nd cycle after 8 m, + AZA maintenance therapy 150 mg qd	MTX maintenance therapy 15 mg qw 7 m after Dx	7.5 mg qd	Progression of ILD, relapsing myositis	Improvement of ILD and lung function
RTX 3	AZA maintenance therapy 150 mg qd	RTX remission induction 2 × 1 g 19 m after Dx	7.5 mg qd	Progression of ILD, relapsing myositis	Relapse and worsening of DLCO, stable FVC
RTX 4	MTX maintenance therapy 15 mg qw + ADA first 3 m	2nd: AZA maintenance therapy 150 mg qd3rd: RTX remission induction 2 × 1 g 35 m	–	Progression of ILD	Progression of ILD, stable lung function
RTX 5	RTX remission induction 2 × 1 g (6 m) after CT2 (25 m after Dx)	AZA maintenance therapy 125 mg qd 28 m after Dx	10 mg qd	Progression of ILD, relapsing myositis	Stable ILD, persistent joint complaints and fatigue
RTX 6	AZA maintenance therapy 100 mg qd	RTX remission induction 2 × 1 g 7 m after Dx	2.5 mg qd	Relapsing arthritis, frequent GC pulses	Clinical and radiographic remission^*^
RTX 7	MMF maintenance therapy 2.5 g qd	RTX remission induction 2 × 1 g 8 m after Dx	5 mg qd	ILD at Dx	Improvement of ILD, remitting flares and bacterial infections
RTX never 1	CYC remission induction first 6 m after Dx	AZA maintenance therapy 150 mg qd	5 mg qd	–	Lost to follow-up
RTX never 2	Mitoxantrone (5 mg/m^2^) multiple sclerosis treatment	–	20 mg qd	–	Lost to follow-up
RTX never 3	GC monotherapy 5 mg first 35 m	MMF maintenance therapy after 41 m (CT2)	5 mg qd	–	Stable ILD
RTX never 4	LEF 20 mg qd + CsA mg/kg 12 m	LEF 20 mg qd + HCQ	5 mg qd	–	Clinical and radiographic remission^*^
RTX never 5	AZA maintenance therapy 100 mg qd	–	10 mg qd	–	Clinical and radiographic remission^*^

* Clinical remission describes the absence of extrathoracic complaints (e.g., myalgia and arthralgia), whereas radiographic remission is defined as the improvement of radiographic findings and stabilization/improvement of PFT.

*AZA, azathioprine; CsA, cyclosporine A; CYC, cyclophosphamide; DLCO, diffusion capacity for carbon monoxide; Dx, diagnosis; FVC, forced vital capacitiy; GC, glucocorticoids; HCQ, hydroxychloroquine; ILD, interstitial lung disease; LEF, leflunomide; m, month(s); MMF, mycophenolate mofetil; MTX, methotrexate; NA, not available; qd, daily; qw, weekly; RTX, rituximab*.

### Pulmonary Function Testing

**Figure 1:**
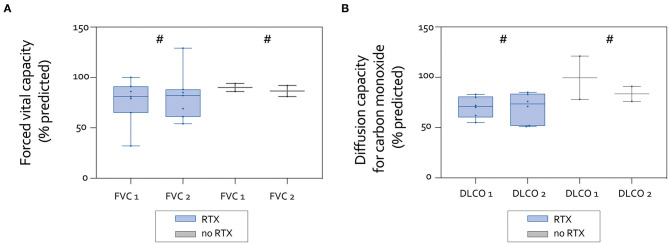
Pulmonary function testing of patients with available data. **(A)** Forced vital capacity (FVC, % predicted) at baseline and follow-up. Spaghetti plots of individual patients (left) and Box-and-Whisker plots with median values (right) did not show statistically significant changes. **(B)** Diffusion capacity for carbon monoxide (DLCO, % predicted) at baseline and follow-up. Spaghetti plots of individual patients (left) and Box-and-Whisker plots with median values (right) did not show statistically significant changes (^#^not significant).

PFTs were obtained at baseline and throughout the course of the study. Forced vital capacity (FVC) and diffusion capacity for carbon monoxide (measured as single-breath carbon monoxide diffusion capacity; DLCO) are presented in Figures 1A,B. Baseline and follow-up PFTs were available for nine (FVC) and eight (DLCO SB) patients. Patients in the RTX group had worse baseline values for FVC and DLCO compared to the non-RTX group. However, the differences were not statistically significant.

### High-Resolution Chest Computed Tomography Findings

HRCT of the chest was performed for each of the 12 patients at baseline (CT1). A second CT scan (CT2) was available in 10 patients after a median time span of 14.5 months (6–72). A third CT scan (CT3) was available in six patients, at a median time of 30 months (17–156) after the first scan.

The interrater agreement κ between the two radiologists was 0.82 for GGO, 0.54 for FIB and 0.05 for HC, corresponding to very good (GGO), moderate (FIB), and poor (HC) agreement, respectively.

**Figure 2:**
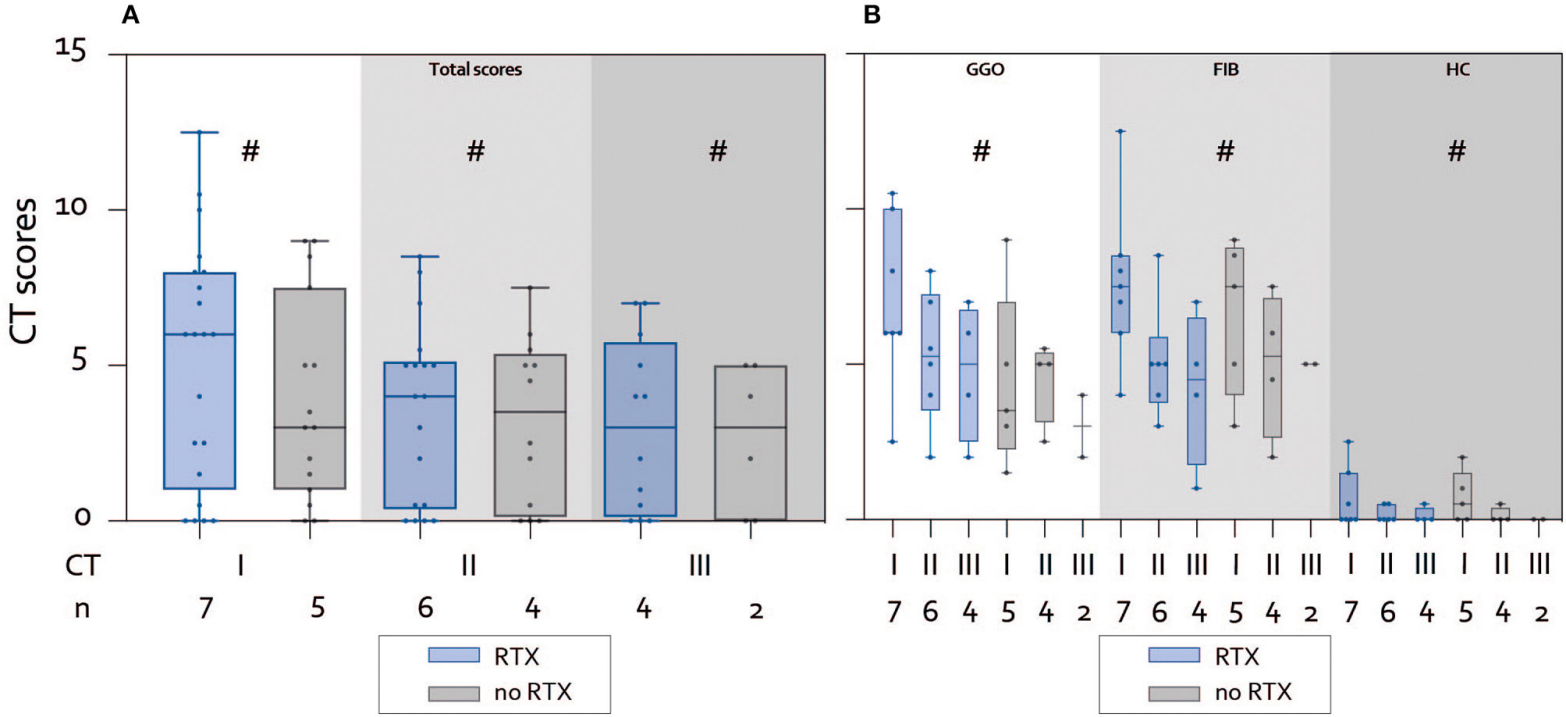
Course of interstitial lung disease changes on chest computed tomography (CT) stratified according to treatment (Rituximab [RTX] vs. no RTX). **(A)** Total CT scores in the whole cohort. Mean CT scores with range for CT 1, 2, and 3 comparing the RTX vs. non-RTX group. There were no statistically significant differences (^#^not significant). **(B)** CT scores stratified according to type of change. Scores for ground glass opacities (GGO), lung fibrosis, interlobular changes and bronchiectasis (FIB), and honey combing (HC) decreased over time without reaching statistical significance (^#^not significant).

Seven patients who received RTX during the study period were compared with five patients without RTX treatment. The total CT scores in the RTX vs. non-RTX groups are presented in Figure 2A and Supplementary Table 1. Overall, the mean CT scores declined over time in both groups, but there were no statistically significant differences neither between groups (RTX vs. no RTX) nor between CTs (CT 1 through CT 3).

Also, the CT scores for the specific findings of GGO, FIB, and HC showed a progressive decrease with treatment over time (Figure 2B). This was observed for patients in the RTX groups and in the non-RTX group from CT 1 through CT 3. However, there were no statistically significant differences between groups nor from CT 1 through CT 3.

### Overall Outcome

No patient died during the study. Radiological findings improved in most patients, exemplified by a decrease of the GGO and FIB scores. We did not observe worsening of the low baseline scores for HC. PFTs were stable or improved in the majority of patients. One patient developed pneumonia and sepsis from a urological source under immunosuppression with the need for hospitalization. No other serious adverse events were documented.

## Discussion

We have shown that pulmonary outcomes, as assessed by PFT and HRCT, did not differ between the varying therapeutic regimens. Nevertheless, the use of RTX was employed in patients with more severe disease as demonstrated by a numerically (although not statistically significant) higher GGO score at baseline, more frequent relapses, and a higher prevalence of myositis.

The prevalence of anti-Jo1 abs in our patient cohort was 83.3%. Anti-SS-A abs can be detected in about half of the patients with ASyS (13); in our cohort, 58.3% of patients tested positive for SS-A abs. Radiological signs of arthritis were present in 58% of the patients. According to the literature, arthritis is the presenting symptom in about 25% of cases of ASyS (15). At least 50–60% of patients with detectable anti-Jo1 and anti-PL-7 abs have clinically active arthritis defined by tenderness or joint swelling in the course of the disease (2, 16). Of these, two thirds have a rheumatoid arthritis-like symmetrical polyarthritis while one third presents with an asymmetrical oligoarthritis (13, 15). Radiographs in patients with ASyS may reveal erosive changes at the wrists, MCP- and PIP-joints, especially in a subset of ASyS patients with positive anti-CCP antibodies (11). Irrespective of the presence of rheumatoid factor or anti-CCP antibodies, ultrasonography can demonstrate severe inflammatory arthritis with erosive RA-like pattern in selected patients with ASyS (17).

Myositis was histologically proven in 41.7% of our patients. Most case series and registries include ASyS patients when myositis becomes clinically apparent or patients initially diagnosed with IIM are diagnosed as ASyS. For this reason, the reported frequency of 75% for myositis in ASyS may be lower early in the course of the disease (1, 13). Moreover, anti-PL7 ab positive patients frequently present an early-onset ILD accompanied by an amyopathic course compared to anti-Jo1 positive patients (18).

Given the rarity of ASyS, little is known about the long-term effects of immunosuppressive therapy on the course of ILD. Lung involvement represents the most serious and life-threatening complication of ASyS, and, therefore, early commencement of an adequate therapy is crucial. The therapeutic response of the disease to immunosuppression can be assessed by the course of the ILD. Normal PFTs at the initiation of treatment are associated with stable or even an improved course of ASyS-ILD, whereas dyspnea and decreasing FVC correlate with a poor prognosis and progression of ILD in ASyS (8). The majority of patients in our cohort had normal baseline PFTs and the data revealed stable lung function in most patients over time. GCs were the most common treatment used, but all except one patient received additional immunosuppressive therapy. The improvement of lung HRCT scores observed in our study indicates a positive response of pulmonary parenchymal abnormalities to immunosuppression: fibrotic changes decreased in about 90% of patients, GGOs improved in about 60% of patients in the second available CT scan. Even if not universally present, HC also improved.

With respect to pulmonary function and overall outcome, RTX is a promising agent, especially early in the course of ASyS-ILD (19), findings which are supportive of our data. The majority of patients who received RTX in our study had clinically apparent myositis, more frequently had arthritis, or had relapsing disease with another immunosuppression.

In two of seven cases treated with RTX, the drug was started as a first-line therapy due to an early manifestation of ASyS-ILD. Additionally, our data demonstrate that other immunosuppressive agents also lead to improved HRCT imaging findings over time, but these patients differed in terms of disease severity and extrapulmonary manifestations.

The limitations of our study include its retrospective design and the small sample size lacking a control group. The intervals between follow-up HRCT scans varied considerably. Although we only examined patients with the presence of ILD at baseline (the first available HRCT), a clinically apparent lung disease may not have been evident in all patients at first presentation but may have developed over time. Abnormal radiology findings indicate an active underlying pulmonary disease which has not yet become apparent clinically. Consequently, the effect of immunosuppressive treatment must be interpreted with these limitations in mind. Also, we did not assess dyspnea scores, such as the St. George's respiratory questionnaire, in all patients since these are not consistently performed routinely in non-pulmonary clinics. The interrater agreement showed a moderate discrepancy in the assessment of HC. This has also been observed other studies, such as the Scleroderma Lung Study (14). However, as outlined above, HC was only rarely present. We, therefore, feel that this discrepancy does not severely affect our conclusions.

In conclusion, our results indicate a trend toward an improvement of ASyS-ILD outcome under treatment with RTX and other immunosuppressive therapies as well as a stabilization of PFT. RTX seems to be superior in patients with a higher number of clinical manifestations, including ASyS-ILD, myositis, arthritis, and in patients with relapsing disease. Nevertheless, prospective trials with pre-specified endpoints are required to further elucidate the impact of immunosuppression on progression and outcome of ASyS-ILD.

## Data Availability Statement

The original contributions presented in the study are included in the article/Supplementary Materials, further inquiries can be directed to the corresponding author/s.

## Ethics Statement

The studies involving human participants were reviewed and approved by the ethics committee of the University Medical Center Goettingen (Protocol no. 4/8/19). All patients consented to the use of their routinely collected data for research purposes.

## Author Contributions

PK conceived the study, treated patients, abstracted and analyzed data, created the figures, and wrote the manuscript. JG-R abstracted and analyzed data, created the tables, and co-wrote the manuscript. LR abstracted data, reviewed and revised the manuscript critically. EM-S scored imaging data, contributed to the methodology, and revised the manuscript critically. UO analyzed data and revised the manuscript. CS and JS treated the patients, analyzed data, and critically revised the manuscript. JL scored imaging data, contributed figures, and revised the manuscript critically. RV conceived the study, analyzed data, co-wrote, and revised the manuscript critically. All authors contributed to the article and approved the submitted version.

## Conflict of Interest

PK has received honoraria and travel support by Abbvie, Bristol-Myers-Squibb, Chugai, Gilead, Glaxo Smith Kline, Janssen-Cilag, Pfizer, and Sanofi-Aventis, all unrelated to this study. JG-R has received travel support by Abbvie and Janssen-Cilag, unrelated to this study. JS has received payments for advisory boards, honoraria, travel expenses, and research projects from Alnylam, Bayer, Biogen, BioMarin, Biotest, CSL Behring, Grifols, LFB, Novartis, Octapharma, Pfizer, all unrelated to this study. JL reported honoraria from Toshiba Medical Systems and Bayer Pharma AG as well as travel support from Boston Scientific, all unrelated to this study. RV has received honoraria and travel support by Abbvie, Janssen-Cilag, Pfizer and Sanofi-Aventis, all unrelated to this study. The remaining authors declare that the research was conducted in the absence of any commercial or financial relationships that could be construed as a potential conflict of interest.

## References

[1] Solomon J Swigris JJ Brown KK. Myositis-related interstitial lung disease and antisynthetase syndrome. J Bras Pneumol. (2011) 37:100–9. 10.1590/S1806-37132011000100015 21390438 PMC3676869

[2] Chatterjee S Prayson R Farver C. Antisynthetase syndrome: not just an inflammatory myopathy. Cleve Clin J Med. (2013) 80:655–66. 10.3949/ccjm.80a.12171 24085811

[3] Marguerie C Bunn CC Beynon HL Bernstein RM Hughes JM So AK. Polymyositis, pulmonary fibrosis and autoantibodies to aminoacyl-tRNA synthetase enzymes. Q J Med. (1990) 77:1019–38. 10.1093/qjmed/77.1.1019 2267280

[4] Schmidt J. Current classification and management of inflammatory myopathies. J Neuromuscul Dis. (2018) 5:109–29. 10.3233/JND-180308 29865091 PMC6004913

[5] Stuhlmüller B Schneider U González-González J-B Feist E Disease specific autoantibodies in idiopathic inflammatory myopathies. Front Neurol. 10:438. 10.3389/fneur.2019.00438 PMC6519140 31139133

[6] Monti S Montecucco C Cavagna L. Clinical spectrum of anti-Jo-1-associated disease. Curr Opin Rheumatol. (2017) 29:612–7. 10.1097/BOR.0000000000000434 28796005

[7] Marie I Hatron P-Y Cherin P Hachulla E Diot E Vittecoq O. Functional outcome and prognostic factors in anti-Jo1 patients with antisynthetase syndrome. Arthritis Res Ther. (2013) 15:R149. 10.1186/ar4332 24286268 PMC3978997

[8] Trallero-Araguás E Grau-Junyent JM Labirua-Iturburu A García-Hernández FJ Monteagudo-Jiménez M Fraile-Rodriguez G. Clinical manifestations and long-term outcome of anti-Jo1 antisynthetase patients in a large cohort of Spanish patients from the GEAS-IIM group. Semin Arthritis Rheum. (2016) 46:225–31. 10.1016/j.semarthrit.2016.03.011 27139168

[9] Cavagna L Monti S Caporali R Gatto M Iaccarino L Doria A. How I treat idiopathic patients with inflammatory myopathies in the clinical practice. Autoimmun Rev. (2017) 16:999–1007. 10.1016/j.autrev.2017.07.016 28778704

[10] Cavagna L Caporali R Abdì-Alì L Dore R Meloni F Montecucco C. Cyclosporine in anti-Jo1-positive patients with corticosteroid-refractory interstitial lung disease. J Rheumatol. (2013) 40:484–92. 10.3899/jrheum.121026 23418387

[11] Meyer A Lefevre G Bierry G Duval A Ottaviani S Meyer O. In Antisynthetase syndrome, ACPA are associated with severe and erosive arthritis: an overlapping rheumatoid arthritis and antisynthetase syndrome. Medicine. (2015) 94:e523. 10.1097/MD.0000000000000523 25997035 PMC4602869

[12] Korsten P Rademacher J-G Seitz CS Zschüntzsch J Mößner R Zeisberg M Interdisziplinäre Fallkonferenzen als Chance für Myositis-Patienten? Nervenheilkunde. (2019) 38:377–80. 10.1055/a-0884-5633

[13] Cavagna L Nuño L Scirè C Govoni M Longo FJ Franceschini F. Clinical spectrum time course in anti Jo-1 positive antisynthetase syndrome: results from an international retrospective multicenter study. Medicine. (2015) 94:e1144. 10.1097/MD.0000000000001144 26266346 PMC4616698

[14] Goldin J Elashoff R Kim HJ Yan X Lynch D Strollo D. Treatment of scleroderma-interstitial lung disease with cyclophosphamide is associated with less progressive fibrosis on serial thoracic high-resolution CT scan than placebo: findings from the scleroderma lung study. Chest. (2009) 136:1333–40. 10.1378/chest.09-0108 19892673 PMC2773360

[15] Cavagna L Nuño L Scirè CA Govoni M Longo FJL Franceschini F. Serum Jo-1 autoantibody and isolated arthritis in the antisynthetase syndrome: review of the literature and report of the experience of AENEAS Collaborative Group. Clinic Rev Allerg Immunol. (2017) 52:71–80. 10.1007/s12016-016-8528-9 26782036

[16] Cavagna L Trallero-Araguás E Meloni F Cavazzana I Rojas-Serrano J Feist E. Influence of antisynthetase antibodies specificities on antisynthetase syndrome clinical spectrum time course. J Clin Med. (2019) 8:2013. 10.3390/jcm8112013 31752231 PMC6912490

[17] Miller JB Danoff SK Bingham CO Paik JJ Mecoli CA Tiniakou E. Sonographic findings from inflammatory arthritis due to antisynthetase syndrome. Clin Rheumatol. (2019) 38:1477–83. 10.1007/s10067-019-04471-y 30810913 PMC6541484

[18] Fischer A Swigris JJ du Bois R Lynch D Downey GP Cosgrove GP. Anti-synthetase syndrome in ANA and anti-JO-1 negative patients presenting with idiopathic interstitial pneumonia. Respir Med. (2009) 103:1719–24. 10.1016/j.rmed.2009.05.001 19497723 PMC2857337

[19] Andersson H Sem M Lund MB Aaløkken TM Günther A Walle-Hansen R. Long-term experience with rituximab in anti-synthetase syndrome-related interstitial lung disease. Rheumatology. (2015) 54:1420–8. 10.1093/rheumatology/kev004 25740830

