# Supplementary material for: Antisynthetase Syndrome-Associated Interstitial Lung Disease: Monitoring of Immunosuppressive Treatment Effects by Chest Computed Tomography
Source: Front Med (Lausanne). 2021 Jan 25;7:609595. doi: 10.3389/fmed.2020.609595 (PMC7868424; doi:10.3389/fmed.2020.609595)
Supplement: Supplementary file 1 [file Data_Sheet_1.DOCX]

# Antisynthetase syndrome-associated interstitial lung disease: monitoring of immunosuppressive treatment effects by chest computed tomography

**Peter Korsten^1*#^, Jan-Gerd Rademacher^1#^, Linn Riedel^1^, Eva-Maria Schnitzler^2^, Ulrike Olgemöller^3^, Cornelia Sabine Seitz^4^, Jens Schmidt^5^, Jörg Larsen^2^, Radovan Vasko^1^**

^1^Department of Nephrology and Rheumatology, University Medical Center Goettingen, Goettingen, Germany ^2^Institute of Diagnostic and Interventional Radiology, University Medical Center Goettingen, Goettingen, Germany ^3^Department of Cardiology and Pulmonology, University Medical Center Goettingen, Goettingen, Germany ^4^Department of Dermatotology, Allergology, and Venereology, University Medical Center Goettingen, Goettingen, Germany ^5^Department of Neurology, University Medical Center Goettingen, Goettingen, Germany

^#^these authors contributed equally

*** Correspondence:**Dr. Peter Korsten
peter.korsten@med.uni-goettingen.de

**Supplementary Information**

**
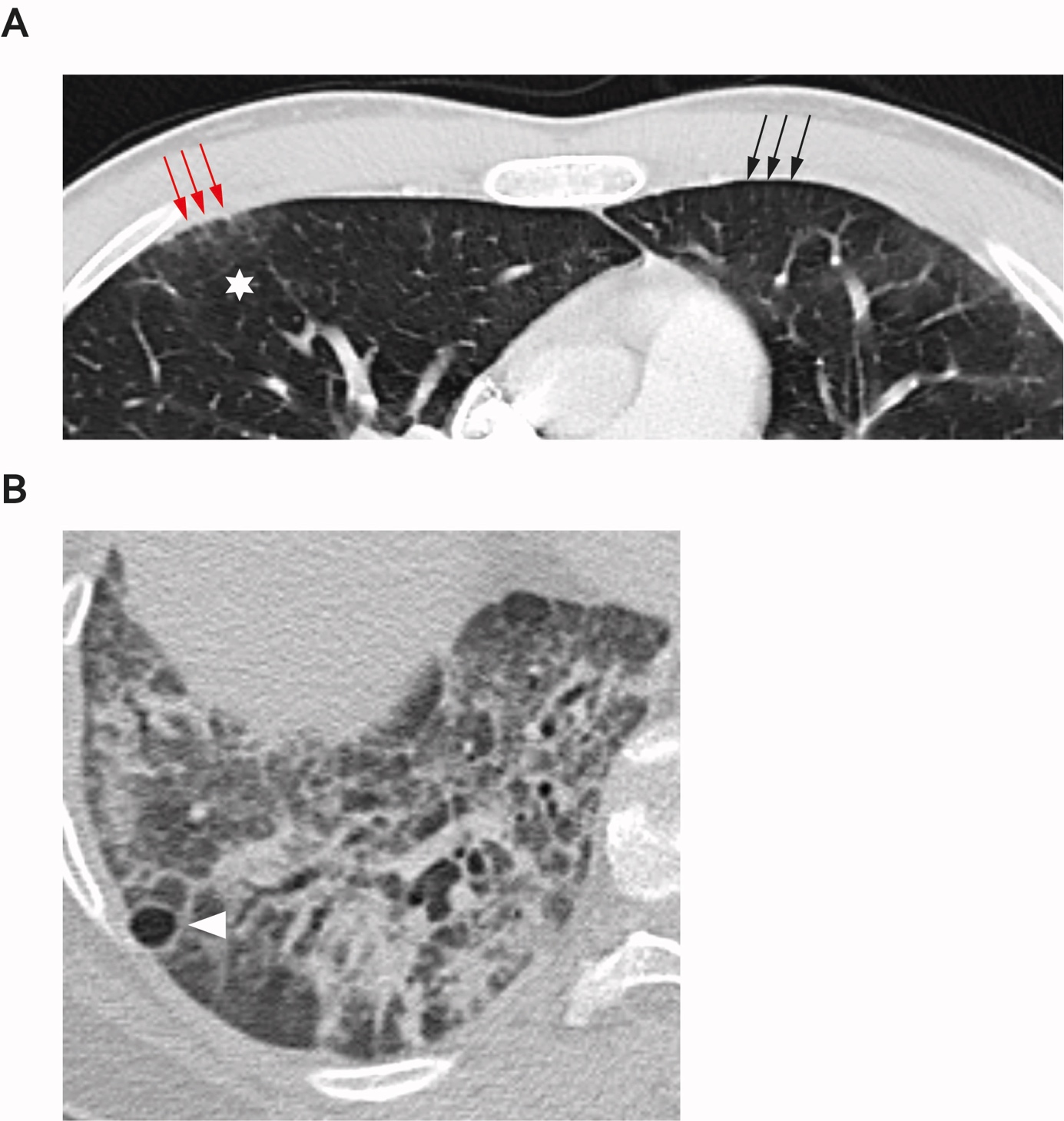
Figure S1. Illustration of high-resolution computed tomography terms used.**

Ground-glass opacification (GGO), constituting a milky increase in parenchymal density, preserving the contrast to neighboring vasculature (**A**, asterisk), reticulation (FBI), characterized by abnormally thickened interlobular septations with a net-like appearance (**A**, red arrows) and honeycombing (HC), defined by subpleural cysts with thickened walls, only a few mm in size (**B**, arrowhead). Please note normal, not thickened interlobular septa in the opposite lung (**A**, black arrows).

**
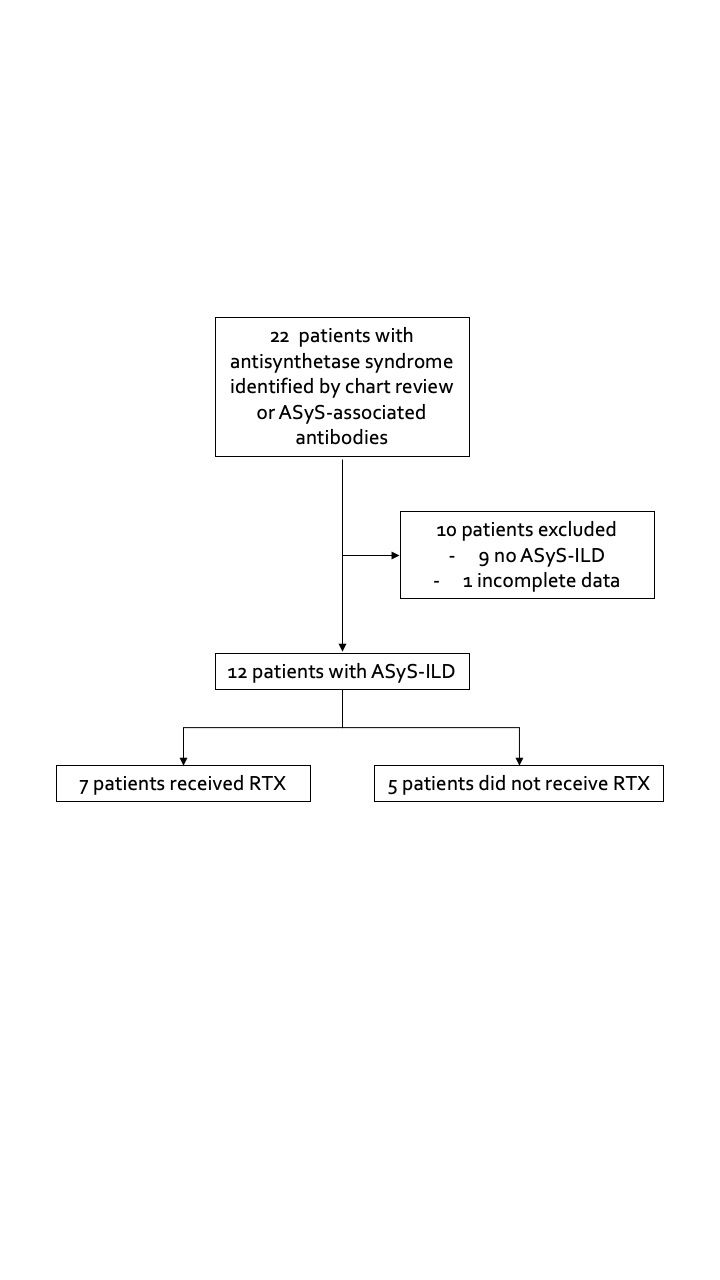
**

**Figure S2. Flowchart of patient selection.**

**Abbreviations:** ASyS, antisynthetase syndrome; ILD, interstitial lung disease; RTX, rituximab.

**Supplementary Table S1: Pulmonary function test and radiologic scores RTX ever vs. RTX never at baseline and follow-up**

|  |  |  | **RTX ever** | | R**TX never** | |
| --- | --- | --- | --- | --- | --- | --- |
|  |  |  | median | n | median | n |
| **PFT** | **FVC** | Baseline | 81% (32 - 100) | 7 | 90% (86 - 94) | 2 |
|  |  | Follow up | 82% (54 - 129) | 7 | 86.5% (81 - 92) | 2 |
|  | **DLCO** | Baseline | 84.5% (59 - 98) | 6 | 114.5% (93 - 136) | 2 |
|  |  | Follow up | 84.5% (71 - 95) | 6 | 111 | 1 |
| **Radiologic score** | | Baseline | 14 (10 - 25) | 7 | 11.5 (6.5 - 17.5) | 5 |
|  |  | Follow-up  (last CT) | 9.25 (7 - 11) | 6 | 9.75 (7 - 12.5) | 4 |

**Abbreviations:** CT, computed tomography; DLCO, diffusing capacity of the lung for carbon monoxide; FVC, forced vital capacity; PFT, pulmonary function test; RTX, rituximab.
